# Diffuse Arterial Thrombosis as a First Manifestation of Occult Malignancy

**DOI:** 10.1155/2016/1658392

**Published:** 2016-10-05

**Authors:** Marija Vavlukis, Irina Kotlar, Emilija Chaparoska, Emilija Antova, Sasko Kedev

**Affiliations:** University Clinic of Cardiology, University of Saints Cyril and Methodius, Skopje, Macedonia

## Abstract

*Aim*. To raise the awareness of a hypercoagulability state as it is often associated with the different types of malignancies. Venous thromboembolism is a frequent complication in these patients, and usually it happens after the diagnosis of cancer is confirmed. However, hypercoagulability disorders presenting as the first symptoms or signs in the cancer patients have rarely been reported. Furthermore, arterial thrombosis is extremely rare even in cancer patients.* Method*. Review of the case characteristics and literature review.* Results*. We present a case of 39-year-old woman who was admitted to our hospital because of intermittent claudication in the right lower extremity. CT angiography revealed multiple thrombi in the arterial system starting from the left ventricle, followed by a thrombus in the distal part of the descending aorta, in the superior mesenteric artery, and in the right popliteal artery. Further investigation of this young patient with no risk factors for hypercoagulable state and no other comorbidities led to complete work-up including diagnostic evaluation for malignancy. The suspicion was confirmed after performing upper endoscopy with biopsy, which revealed malignant neoplasm of the stomach.* Conclusion*. Whenever a patient suffers hypercoagulability disorders, even arterial thrombosis, we should always consider the possibility of a cancer.

## 1. Introduction

Thromboembolic complications affect approximately 15% of the cancer patients [1]. The oldest theory of this hypercoagulable state was established 150 years ago, when Trousseau in 1865 first described migratory thrombosis as the first manifestation of occult gastric cancer [2]. Today this concept of “Trousseau's syndrome” is used to describe not only migratory thrombosis that precedes the diagnosis of occult cancer but also any “hypercoagulable state associated with malignant cancer” [3, 4].

Although the underlying biochemical mechanisms are poorly understood they are likely to be multiple and, probably, synergistic [5]. It is believed that this hypercoagulable or prothrombotic state of malignancy occurs due to the ability of tumor cells to activate the coagulation system [6]. The cancer cells have the ability to produce and secrete procoagulant/fibrinolytic substances and inflammatory cytokines, and the interaction between tumor cells and blood cells (monocytes, platelets, and neutrophils) or vascular cells can activate the coagulation cascade. Other mechanisms of thrombus promotion in malignancy include nonspecific factors such as the generation of acute phase reactants and necrosis, abnormal protein metabolism (i.e., paraproteinemia), and hemodynamic compromise (i.e., stasis) [6]. As a paraneoplastic syndrome, this disorder is known to be frequently associated with a variety of malignancies, including the ones that are affecting the gastrointestinal system.

Deep vein thrombosis (DVT) and pulmonary embolism (PE) are the two most common thromboembolic complications in cancer [7], but arterial thrombosis in the set of secondary hypercoagulable state due to malignancy is extremely rare [8]. The percent of the cases where arterial thrombosis is a complication of this hypercoagulability remains unknown.

The large proportion of terminal events in neoplastic diseases is thrombotic, leading to the hypothesis that cancer is a prothrombotic disease [5]. Furthermore, many of the standard chemotherapies in cancer are prothrombotic. Accordingly, thromboprophylaxis in cancer with heparins or oral anticoagulation (such as warfarin), especially in high risk groups (such as those who are immobile and on high doses of chemotherapy), may be an important therapy [5]. We are describing such a clinical case.

## 2. Case Presentation

Medical history is as follows: 39-years-old woman was hospitalized in our clinic with signs of intermittent claudication (Fountain IIb) in the right lower extremity. The symptoms started 15 days prior to admission and were accompanied with unpleasant tingling sensation in the leg. The patient sought medical help in a hospital facility, but no extensive diagnostic and therapeutic procedures were undertaken, and she was recommended to contact a cardiologist to perform Doppler ultrasound of the lower limbs. Although the general aspect of the patient alluded to a healthy individual, a more detailed history uncovered severe weight loss of 15 kg in the last 5 months. She had no risk factors for atherosclerosis and has never been hospitalized prior to this event. Physical examination revealed a cold, marmorized right lower extremity with an absent pulse starting at the level of the right popliteal artery. The laboratory analyses revealed sideropenic anemia (hemoglobin of 9.8 g/dL and mean corpuscular volume of 73 fL) with high erythrocyte sedimentation rate (66/120), elevated C reactive protein (118 mg/L), and elevated D-dimers (1.10 micrgr/mL). Other biochemical parameters were within normal range. The echocardiographic examination visualised large mobile structure in the left ventricle attached to the lateral wall with dimensions 24 × 14 mm with echocardiographic characteristics typical of thrombotic material.

**Figure 1:**
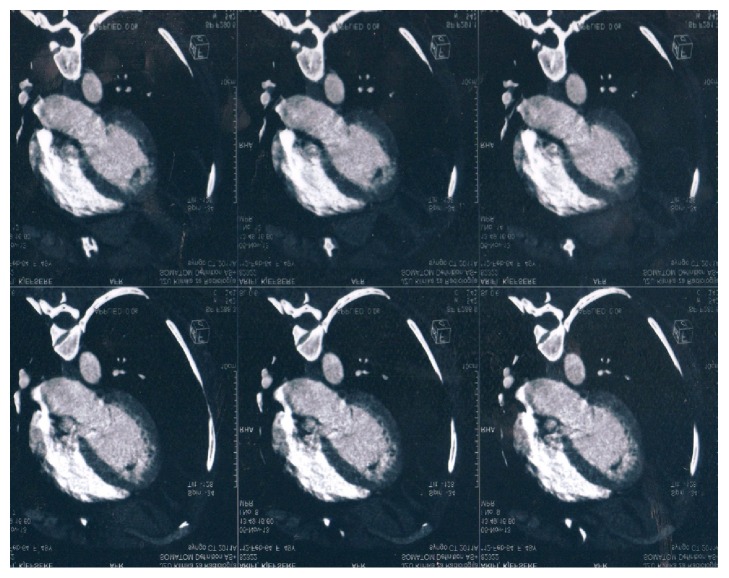
Left ventricular thrombus.

**Figure 2:**
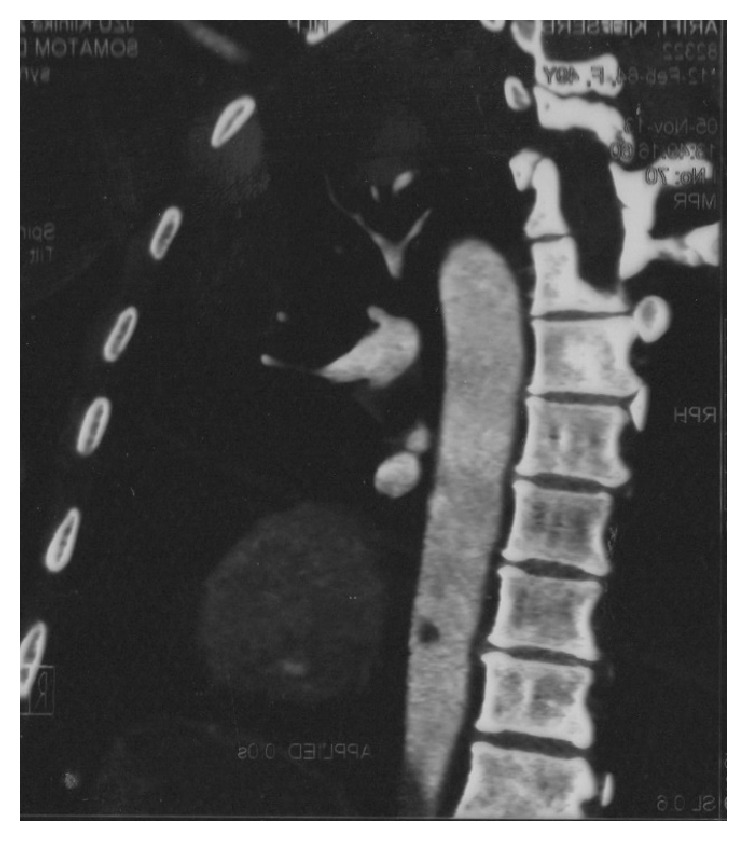
Thrombus in the descending aorta.

**Figure 3:**
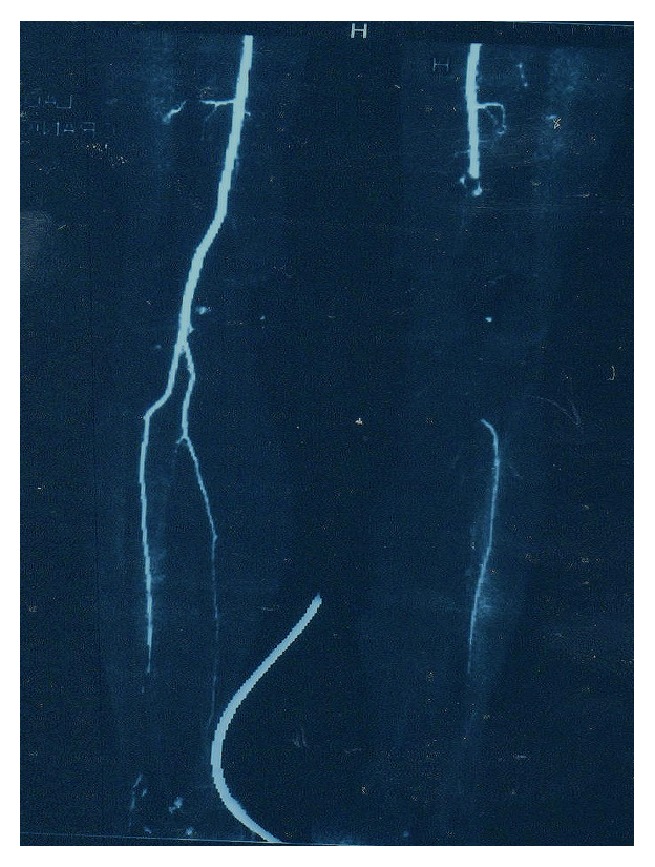
Thromboembolism leading to occlusion of the right popliteal artery.

The possibility of arterial thrombosis led to the further diagnostic work-up towards CT angiography. It revealed multiple arterial thrombi starting with a big floating thrombus in the left ventricle (Figure 1), followed by intramural thrombus in the distal descending aorta (with diameter of 9 mm) (Figure 2) and thrombus in the superior mesenteric artery and the right popliteal artery (Figure 3). The CT scan also incidentally discovered the presence of enlarged lymph nodes in the paraaortic (with largest diameter of 10 mm) and gastroepiploic region. After a consultation with a vascular surgery team and a given recommendation for conservative treatment, the patient was treated with UFH intravenously for 9 days and then overlap and finally switched to warfarin. 11 days later, the control echocardiogram did not show any residual thrombotic material in the left chamber. The control hemostasis was in the normal range, with international normalized ratio (INR) kept in the range of 2-3. The presence of abdominal lymphadenopathy accompanied with hypercoagulable state of unknown origin, severe weight loss, and sideropenic anemia with high sedimentation rate raised the suspicion of undiscovered malignancy. An upper endoscopy was performed and it discovered ulcer like excavation with irregularly formed edges located on the lesser curvature with dimension of 30 mm and three slightly elevated lesions in the proximal part of the gastric corpus. The macroscopic features of these changes were highly suspicious for gastric carcinoma, and the diagnosis was confirmed with biopsy. It revealed the presence of moderately differentiated invasive gastric adenocarcinoma with malignant cells with nuclear grade 2. Immunohistochemical analysis was also performed (HER2/neu(+)). The biopsy suggested an intestinal type of cancer according to Lauren's classification of gastric cancer. After consultation with abdominal surgeon, our patient was directed to the Digestive Surgery Department for estimation of future treatment of the underlying condition.

## 3. Discussion

Thromboembolic disease affects about 15% of cancer patients and remains a challenge for modern medicine for both prophylaxis and treatment [1]. The association of malignancy and hypercoagulable state has been researched for decades but the biochemical mechanisms are not yet fully understood [7]. There is evidence that certain cancers are more likely to be prothrombotic, and this is likely to be influenced by disease staging and bedrest, as well as therapeutic intervention [6]. The current findings indicate that tumor cells are directly prothrombotic and have the ability to produce and secrete procoagulant/fibrinolytic substances and inflammatory cytokines; on the other hand the normal host cells may be also considered as a second response against the cancer cells [5, 6]. Additionally increased levels of coagulation factors, blood turbulence, coexisting inflammatory diseases, use of growth factors, chemotherapy, and irradiation therapy may all induce thrombosis [5]. Examples of molecules elaborated by cancer cells that can predispose to disordered coagulation include tissue factor, a vitamin K-dependent cysteine protease that activates factor X, and a mucin procoagulant that activates prothrombin [9]. This state is the second leading cause of death for cancer patients although, in many of these patients, it represents only one of the many complications of the end-stage disease [10]. A hypercoagulable state related to a cancer is a well-known risk factor for venous thrombosis, but there are fewer data on arterial thrombosis [9]. Arterial thrombosis as a first manifestation of cancer is extremely rare. Javid et al. made a study on 20 patients with malignancy and arterial thrombosis and the most common malignancy was breast cancer [11]. Rigdon [12] reported three patients who developed acute arterial thrombosis in extremities without significant atherosclerosis in arms or legs but who were subsequently discovered to have an occult malignancy (none of these patients were diagnosed with gastric adenocarcinoma). In our case, our patient had symptoms for 5 months before admission but she did not consult a physician due to the uncharacteristic nature of the symptoms. The diffuse thromboembolism, which affected only the arterial system, led to a broad diagnostic work-up for hypercoagulable state, but the laboratory findings, the medical history of weight loss, and the incidentally discovered lymphadenopathy were of great importance for further evaluation in direction of occult malignancy. The upper endoscopy and the biopsy confirmed the suspicion and the patient was treated with anticoagulant therapy. Nowadays recommendations suggest that these patients should receive an anticoagulation therapy [8]. Surgical thrombectomy has an indication in selected cases of recurrent embolism or persistent thrombus despite proper anticoagulation. Patients with a threatened extremity should be treated with emergent surgical revascularization to resolve the symptoms of acute distal embolization, in addition to perioperative intravenous heparin therapy with conversion to oral anticoagulation [8].

## 4. Conclusion

Arterial thrombosis in patients without atherosclerosis and known CVD risk factors should always raise the suspicion of occult malignancy. Complete diagnostic investigation is obligatory and if no other reason for hypercoagulability state could be found screening for malignant cause should be undertaken.

## References

[1] Green K. B., Silverstein R. L. (1996). Hypercoagulability in cancer. *Hematology/Oncology Clinics of North America*.

[2] Trousseau A. (1865). *Phlegmasia Alba Dolens: Clinique Medicale de l-Hotel-Dieude Paris*.

[3] Varki A. (2007). Trousseau's syndrome: multiple definitions and multiple mechanisms. *Blood*.

[4] Falanga A. (2011). The cancer-thrombosis connection. *The Hematologist*.

[5] Blann A. D., Dunmore S. (2011). Arterial and venous thrombosis in cancer patients. *Cardiology Research and Practice*.

[6] Caine G. J., Stonelake P. S., Lip G. Y. H., Kehoe S. T. (2002). The hypercoagulable state of malignancy: pathogenesis and current debate. *Neoplasia*.

[7] Dipasco P. J., Misra S., Koniaris L. G., Moffat F. L. (2012). The thrombophilic state in cancer part II: cancer outcomes, occult malignancy, and cancer suppression. *Journal of Surgical Oncology*.

[8] Okamoto R., Sawai T., Takasaki A. (2013). A case of aortic thrombosis and embolism preceding the progression of early esophageal cancer. *Journal of Cardiology Cases*.

[9] Letai A., Kuter D. J. (1999). Cancer, coagulation, and anticoagulation. *The Oncologist*.

[10] Donati M. B. (1994). Cancer and thrombosis. *Haemostasis*.

[11] Javid M., Magee T. R., Galland R. B. (2008). Arterial thrombosis associated with malignant disease. *European Journal of Vascular and Endovascular Surgery*.

[12] Rigdon E. E. (2000). Trousseau's syndrome and acute arterial thrombosis. *Cardiovascular Surgery*.

